# Experimental Evaluation of UWB Indoor Positioning for Sport Postures

**DOI:** 10.3390/s18010168

**Published:** 2018-01-09

**Authors:** Matteo Ridolfi, Stef Vandermeeren, Jense Defraye, Heidi Steendam, Joeri Gerlo, Dirk De Clercq, Jeroen Hoebeke, Eli De Poorter

**Affiliations:** 1 imec, IDLab, Department of Information Technology, Ghent University, 9000 Gent, Belgium; Jense@Defraye.be (J.D.); jeroen.hoebeke@ugent.be (J.H.); eli.depoorter@ugent.be (E.D.P.); 2 Department of Telecommunications and Information Processing, Ghent University, 9000 Gent, Belgium; Stef.Vandermeeren@UGent.be (S.V.); Heidi.Steendam@UGent.be (H.S.); 3 Department of Movement and Sports Sciences, Ghent University, 9000 Gent, Belgium; Joeri.Gerlo@UGent.be (J.G.); Dirk.DeClercq@UGent.be (D.D.C.)

**Keywords:** UWB, indoor localization, tracking, particle filter, Kalman filter, sports, athletes

## Abstract

Radio frequency (RF)-based indoor positioning systems (IPSs) use wireless technologies (including Wi-Fi, Zigbee, Bluetooth, and ultra-wide band (UWB)) to estimate the location of persons in areas where no Global Positioning System (GPS) reception is available, for example in indoor stadiums or sports halls. Of the above-mentioned forms of radio frequency (RF) technology, UWB is considered one of the most accurate approaches because it can provide positioning estimates with centimeter-level accuracy. However, it is not yet known whether UWB can also offer such accurate position estimates during strenuous dynamic activities in which moves are characterized by fast changes in direction and velocity. To answer this question, this paper investigates the capabilities of UWB indoor localization systems for tracking athletes during their complex (and most of the time unpredictable) movements. To this end, we analyze the impact of on-body tag placement locations and human movement patterns on localization accuracy and communication reliability. Moreover, two localization algorithms (particle filter and Kalman filter) with different optimizations (bias removal, non-line-of-sight (NLoS) detection, and path determination) are implemented. It is shown that although the optimal choice of optimization depends on the type of movement patterns, some of the improvements can reduce the localization error by up to 31%. Overall, depending on the selected optimization and on-body tag placement, our algorithms show good results in terms of positioning accuracy, with average errors in position estimates of 20 cm. This makes UWB a suitable approach for tracking dynamic athletic activities.

## 1. Introduction

As part of one of the biggest revolutions of the digital era, billions of heterogeneous devices are connected through the so-called Internet of things (IoT). Thus, location-based information is becoming more and more relevant [1]. Accuracy is the main evaluation metric for estimated positions. Irrespective of the application domain, it is fundamental to obtain precise information in terms of relative or absolute position. However, not every system has the same requirements and needs [2]. For example, the tracking of animals in stables allows farmers to determine the animals’ habits and provides the possibility of detecting problems through changes in mobility patterns [3]. For most farming applications, cost and energy consumption are the primary optimization criteria, whereas for sport activities, reliability and location accuracy are often considered more critical. Accurate position information can be important for optimizing the athlete’s performance by monitoring the velocity, acceleration, and taken path. In sporting environments, the tracking of athletes has gained much popularity. Even when trying to locate fast moving nodes, there are divergent needs and approaches. For example, during sports events such as basketball or football games, the movement dynamics of the athletes result in the need for high update rates for all the estimated positions in order to consistently keep track of the athletes. A different approach is required when accurate data is needed in (semi)laboratory environment evaluations for scientific use. To research various aspects of sport science disciplines, very expensive technologies are typically used. Among other methods, biomechanical movement analysis with state-of-the art three-dimensional (3D) motion capture systems is a practice that renders highly accurate 3D kinematics of the moving person. An example of such a system is deployed at the Sport Science Laboratory—Jacques Rogge of Ghent University [4]. A marker-based motion capture system (MOCAP) is used. It consists of several infrared (IR) cameras [5] in charge of the estimation of the localization of one or more markers placed on human bodies. This kind of system can provide sub-millimeter accuracy. In general, although vision-based solutions tend to be more accurate than others, there are still some drawbacks. The main issue is the cost. Compared to other technologies, the cost of vision-based systems is still high. Another drawback comes from the intrinsic nature of the camera system: the markers must be in the line of sight (LoS) for multiple cameras, otherwise it becomes impossible to compute the position. This can be avoided using more hardware (using more cameras), which results in even higher prices. A cheaper and easier to deploy alternative is represented by another localization category: radio frequency (RF)-based solutions. The most popular technologies are Bluetooth Low Energy (BLE), WiFi, and radio-frequency identification (RFID), which are known for their ease of deployment, although they are less accurate than other technologies [6,7,8]. In the same category but with better localization accuracy, we find ultra-wide band (UWB). UWB can achieve 10-cm accuracy in LoS conditions and it keeps performing well even in multipath environments [9] such as indoor areas, in which walls and ceilings may reflect signals and create multiple delayed copies.

With this paper, we want to investigate how the highly dynamic movements performed by athletes impact the accuracy of UWB indoor localization systems. The first part of our research involves the placement of the tag, which is the entity that the system has to track, i.e., the equivalent of the markers for the camera-based system. This is necessary in order to understand all possible limitations due to the signal attenuation experienced by the tag when placed on a human body. Afterwards, particle and Kalman filter algorithms are designed to estimate the position of the tag. Moreover, to deal with unwanted errors and distortions, we define three advanced optimizations to further improve the localization algorithm in terms of precision and accuracy. These are bias removal, non-line-of-sight (NLoS) detection, and path determination.

The remainder of the paper is organized as follows. Section 2 outlines the related work for athlete tracking using various technologies. Section 3 and Section 4 are dedicated to the evaluation of the best tag position on the body and to the localization algorithm implementation, respectively. In Section 5, the positioning algorithms are used for two large-scale tests, namely for indoor cycling and running. Finally, Section 6 presents the algorithmic optimizations to improve system accuracy. The paper ends with the conclusions in Section 7.

## 2. Related Work

Various types of radio technologies have been used to track athletes and have been gaining popularity in the last few years. Many companies entered the market advertising high accuracy in terms of positioning estimates, which is a fundamental requirement for collecting useful data on athletes’ activities in order to (among others goals) evaluate the performance and design better training programs. These technologies include video-based localization, Global Positioning System (GPS), Bluetooth, RFID, inertial measurement units (IMUs) and UWB. However, it seems that none of these is a market leader or a de facto standard. In some cases this is due to high costs, while in others it is due to poor accuracy. The environment also limits the deployment of some technologies. For example, many sporting activities are performed indoors, where GPS cannot be used. A study on the importance of positioning systems applied in sports can be found in [10]. The authors highlight the critical requirements that technology has to meet in this field, e.g., an around 20-cm accuracy and a sample rate of at least 15 Hz. They also mention UWB as possibly the biggest player in the market. With the recent commercialization of UWB chips by Decawave [11], UWB has gained popularity in the research community, as this technology is able to provide centimeter-level accuracy at an affordable price. In our paper, we focus on UWB positioning, with the primary goal of assessing the possibility of using UWB in dynamic environments such as those related to athletes’ activities. In [12], a market-available product that uses Decawave technology is tested. Their main focus is the study of the feasibility of using UWB to track and locate dynamic users. They claim that the user speed and velocity dramatically affect the precision of the technology when estimating position. Furthermore, they are able to localize a mobile node with accuracy resolution ranging from 15 cm to 50 cm. However, we believe that with the method tested in our paper, this is less the case. The algorithm we designed and its optimizations improve the accuracy even during dynamic activities. Our solution, with an average error of 20 cm, is on average twice as accurate as the one proposed in [12]. More studies on tracking mobile objects/people with UWB can be found in the scientific literature. In [13], time difference of arrival (TDoA) schemes are used to localize elderly people wearing an UWB tag in environments such as hospitals. Although the authors proved that this technology can localize people with an error of less than 1 m, they did not study the impact of different activities on the system accuracy. Moreover, no tracking algorithms are implemented, making this solution suboptimal for highly dynamic scenarios like the ones we want to investigate. In [14], the tracking behavior is taken into account to study the effect of speed and ‘heading’ (where the tag is heading to). Similar to our work, the authors evaluated the dependencies of the tag’s position on a person. They found that placing the tag on top of the head gives the best results. Unfortunately, only two options were studied in their analysis (head and shoulders), leaving space for further research. Moreover, the dynamics of the tag are a primary issue in their work, as the experiments they conducted are mostly related to deterministic paths such as Lego train tracks, which are not realistic scenarios when athletes have to be tracked. In this paper, we evaluate more positions of the tag, and evaluate different postures/activities typical for various sports, in contrast to the work of [14] where constrained paths are used. Understanding the performance of UWB in mobile and unpredictable scenarios is also the main focus in [15]. Analogously to our research, they tested stationary and dynamic cases with the mobile tag placed in different positions on a wheelchair rather than on the body. Their results are in line with our analysis—they show an average horizontal positioning error of 0.37 m with the tag worn in a GPS vest as the best placement. Moreover, they focus on the overall distance covered, which is computed with a combination of the Kalman filter and low-pass filter. Errors in distance estimations are not investigated further, in contrary to our approach since we provide several tools to overcome systematic errors that may affect the system.

## 3. UWB Performance Evaluation for Sporting Activities

The complexity of the application domain, including various unpredictable movements such as crawling, jumping, and running, requires an extensive study of two important factors, i.e., the impact of both the position of the tag on the athlete’s body and the activity he/she is doing. Therefore, we discuss and analyze these two relevant aspects in this section.

In particular, tests have been performed with the commercial UWB chip designed and manufactured by Decawave and integrated into a development board by Pozyx Labs [16]. Experiments were performed in two different environments, i.e., in a 10 × 12 m^2^ room in a residential environment and at the Sport Science Laboratory–Jacques Rogge of Ghent University.

**Figure 1:**
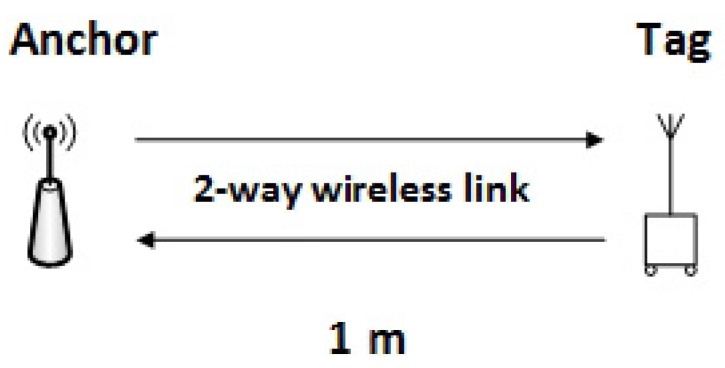
Experimental setup with one anchor and one mobile tag in clear line of sight (LoS).

A first basic setup in the residential room involving one fixed anchor and one tag was used to assess the ranging capabilities of the hardware we used, i.e., to evaluate the UWB accuracy when estimating the distance between the tag and the fixed anchor node (Figure 1).

**Figure 2:**
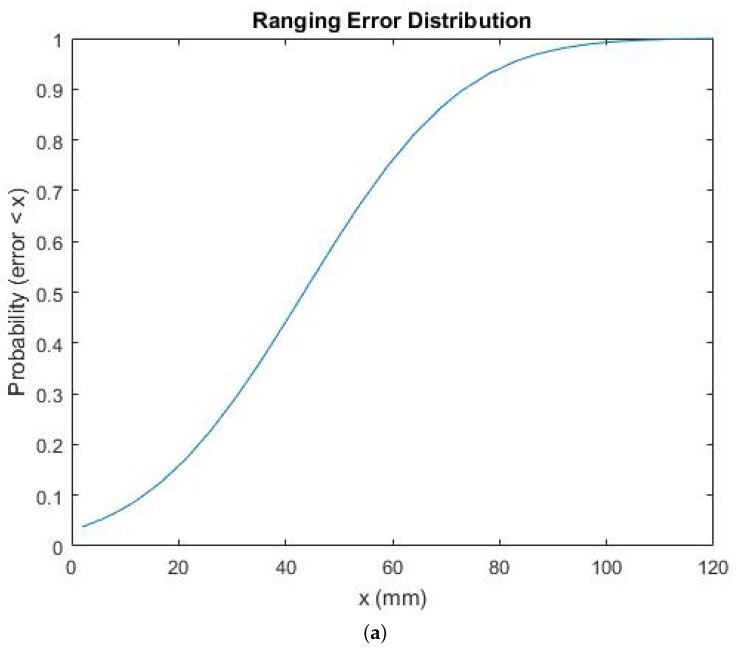

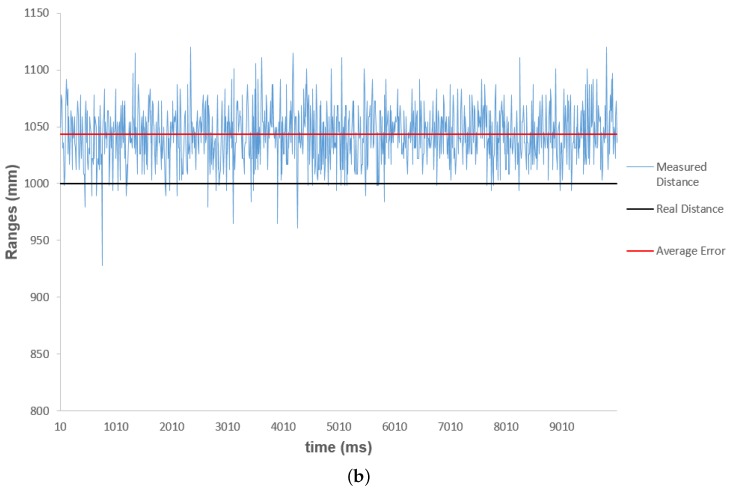
(**a**) Ranging error distribution with one fixed anchor and one tag. (**b**) Estimated ranges over time.

In such an ideal scenario, the results are quite encouraging, as shown in Figure 2a.

The average ranging accuracy is around 4.35 cm, with a standard deviation of 2.33 cm. Note that as the ranging measurements are very stable in time, the error seems to mainly be a systematic error, which we refer to as bias from now on (Figure 2b). This biased behavior is further analyzed and corrected in Section 6.1 of this paper.

### 3.1. Tag Placement Impact

**Figure 3:**
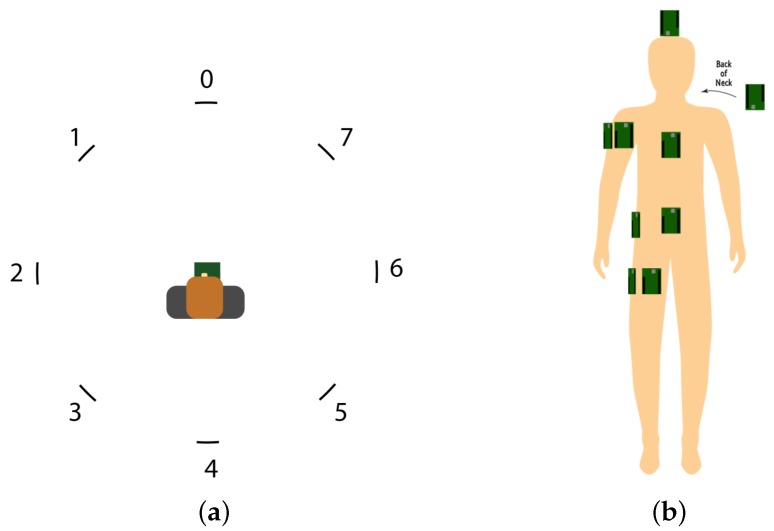
(**a**) Experimental setup with eight fixed anchors and one user equipped with the tag. (**b**) Ultra-wide band (UWB) tag placement on the body.

Having one static tag ranging with only one anchor is rather useless in positioning systems and not realistic when the goal is tracking moving athletes. In realistic conditions, the tag must be able to estimate its distance to multiple anchor nodes in order to calculate its most likely position. Therefore, we now consider the setup of Figure 3a, where multiple anchor nodes are placed in the same room used during the previous experiment. Now, the mobile tag is worn by a user, who is surrounded by eight fixed anchor nodes (numbered from 0 to 7). The tag is in the center of a circle with a 2-m radius. In Table 1, the UWB radio settings used during our measurements are listed.

Ideally, the tag should be able to estimate its distance from as many anchor nodes as possible. However, since the human body influences propagation behavior by absorbing (for the most part) and reflecting (to a lesser extent) part of the radiated waves, some anchor nodes might not be reachable depending on the location of the tag on the body [17]. To evaluate the best possible tag placement on a human body, three metrics are considered , i.e., accuracy, packet loss, and number of visible anchors. It is important to consider all three of these metrics. For example, knowing only the number of available anchors might be indicative of how many are able to successfully communicate with the tag. On the other hand, if the packet loss is the only form of evaluation, it is not possible to know whether sufficient anchors are available for positioning, e.g., three are needed in 2D localization systems.

During the experiments, the tag is placed on nine different parts on the body, namely the front/side of the right arm and the upper legs, chest, neck, stomach, hip, and head. These are also visualized in Figure 3b and listed in Table 1. Table 2 shows that when the tag is positioned on the user’s head, the average packet loss over all anchor nodes is limited to only 1.45% . The behavior becomes more problematic when the tag is placed on the chest. In this case, the UWB signal is obstructed by the human body and in some cases, more than 50% of the ranging packets are lost, e.g., when ranging with anchors 3, 4, and 5. This is due to the orientation of the body, which is positioned looking towards anchor 1.

**Figure 4:**
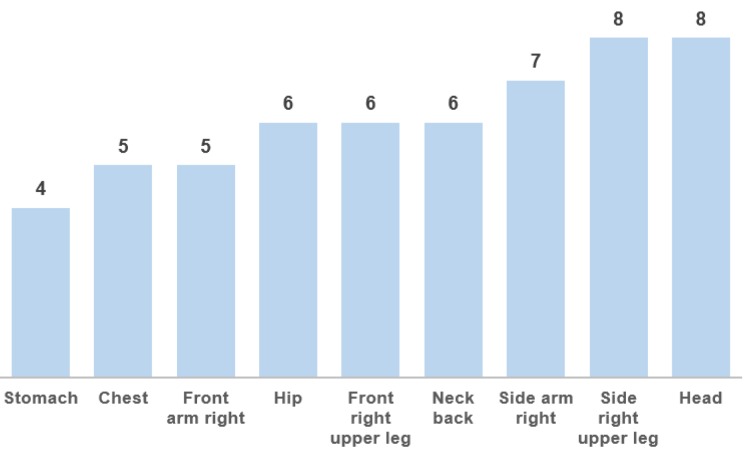
Number of reachable anchors for different on-body tag placement positions.

When assuming that an anchor node is available if the packet loss is <10%, it is possible to calculate the number of anchors available per tag position (see Figure 4). Most tag locations observe at least six anchor nodes, making them suitable for position estimates.

**Figure 5:**
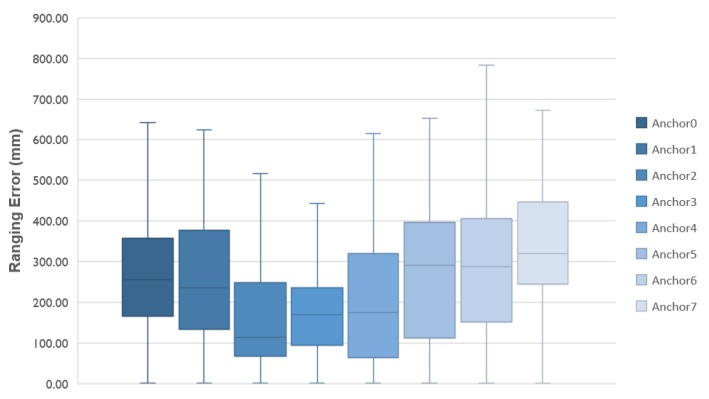
Average ranging error per anchor with the tag placed on the head.

To investigate if all visible anchor nodes are of equal quality, additional accuracy tests are performed with a tag placed on the head. We configure the tag to range with all eight anchors of Figure 3a and we report the results per anchor in Figure 5. If we combine the packet loss (Table 2) with the average ranging error, we notice that high packet loss does not necessarily cause a lower accuracy. For the head case, anchor 4 registers the highest packet loss with failures of 4.8% in the ranging procedure, but it also produces the best results in terms of average ranging error at 16.85 cm (Figure 5).

Finally, for the determination of the optimal tag location on the athletes, the comfort and other aspects such as aerodynamics must also be taken into account. Therefore, in the next paragraph we select three potential placements that performed well based on the metrics we have just discussed, namely the head placement, the neck placement and the side of the arm placement.

### 3.2. Sporting Activity Impact

**Figure 6:**
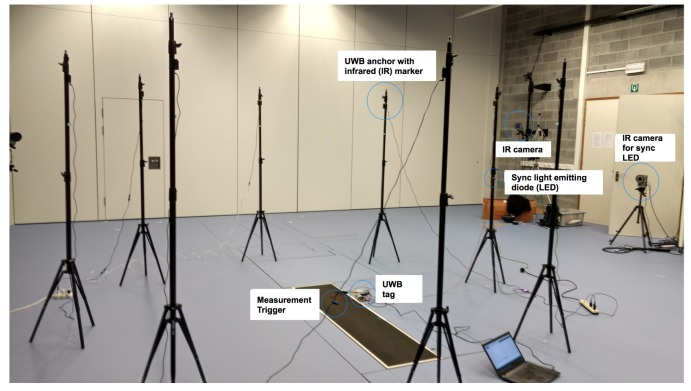
Sport Science Laboratory–Jacques Rogge of Ghent University, wherein eight UWB anchors are installed to position a mobile tag.

For experiments with moving athletes, it is necessary to be able to track the exact position of the on-body tag during the movements. To provide this “gold standard” ground truth of the tag, an advanced marker-based motion capture system (MOCAP) consisting of several IR cameras that can provide sub-millimeter accuracy is used. This MOCAP systems offers millimeter-level accuracy even for fast moving tags. However, one of the limitations of this installation is that the covered volume of the MOCAP system can be increased, but at the cost of lower accuracy. In the interests of scientific accuracy, we opted for a setup with millimeter-level accuracy. As a result, the covered volume is limited to 5 × 5 m^2^, but this permits us to use small 12-mm markers during the experiments, allowing us to locate with millimeter-level accuracy both the anchors and the mobile tag. While the setup of the experiments remains the same as the previous one with eight fixed anchors, the experiments are performed in the Sport Science Laboratory–Jacques Rogge of Ghent University which is equipped with a MOCAP system. In Figure 6, both the lab environment and the equipment used during these tests are shown. First, we want to evaluate the impact on ranging error of various poses (Table 1) while having the tag attached to different parts of the body. More precisely, we focus on four cases: standing, full squat, bent over, and prone on the floor.

**Figure 7:**
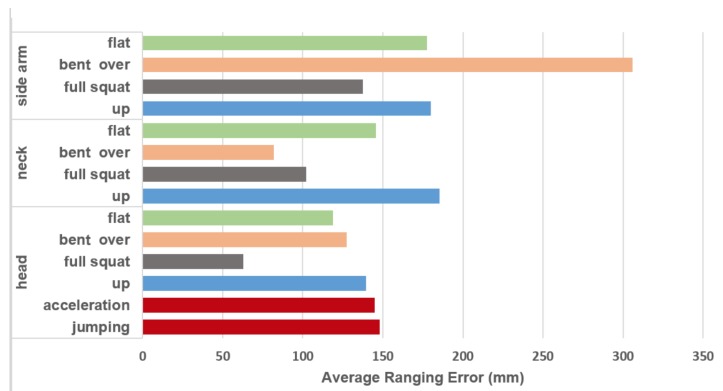
Impact of different postures and activities on ranging accuracy while estimating the distance to eight anchors.

Similarly to what was done before, we collected ranging measurements between the tag and all eight anchor nodes. Afterwards, we processed the data and analyzed the results in terms of accuracy and packet loss, as shown in Figure 7 and Table 3, respectively.

The head case outperforms the others; it has the lowest percentage of failures and the lowest average ranging error. For this reason, we tested two extra activities when the tag was placed on the head, i.e., running with constant acceleration and jumping (Table 1). Although they implicate more failures with respect to the standing still case (from 8% up to 17%), the ranging error is comparable, i.e., 14.5 cm average error for the acceleration scenario, and 14.8 cm and 13.9 cm for the jumping and standing still cases, respectively.

**Figure 8:**
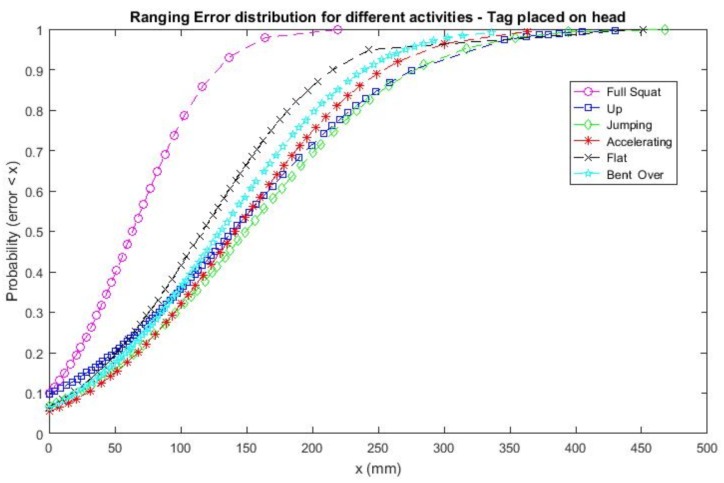
Ranging error distribution for different postures and activities while estimating the distance to eight anchors—head case.

The distribution of the error for the head case is reported in Figure 8. This helps to visualize the impact of different activities and postures. In particular, it is possible to notice that there is not much difference when standing up with respect to the jumping case. However, the antenna surroundings make the mean error slightly different in few regions [18] and therefore, the full squat case seems to have the best ranging accuracy. For the same reason, the flat on the ground posture is the second best case with a cumulative probability of having an accuracy ≤20 cm of 0.85.

## 4. Localization Algorithms

By combining the distance estimations from the previous sections, it is possible to calculate the most likely spatial position of the tag. In this section, we will describe how the position of a person is derived from the UWB measurements using particle and Kalman filters. In the literature, the Kalman and particle filter are frequently used to track an object or person. With these filters it is possible to fuse information from different sensors, e.g., UWB and IMU, and track a mobile user. Drawbacks of the Kalman filter include that it is less suited for non linear systems (such as localization). Therefore, extensions of the Kalman filter, e.g., the extended Kalman filter (EKF) and unscented Kalman filter (UKF), have been developed to better model non-linearities in the system. On the other hand the particle filter is far more capable of handling non-linear systems. However, a disadvantage of the particle filter is that it requires much more computational power. We will compare both Kalman and particle filters. To test our filters, we took additional measurements in the Sport Science Laboratory, where eight UWB anchors were equally spaced on the edge of a 5 × 5 m${}^{2}$ square surface. Four anchors were positioned on the corners of this area and the other four anchors were positioned at the center of each side. The estimated positions are then compared in terms of accuracy with gold standard MOCAP measures.

### 4.1. Particle Filter

A particle filter is an algorithm that can be used to estimate the state of a dynamical system. In this paper, the state $x_{t}$ will be the position and velocity of a person that wears the UWB tag, i.e., $x_{t}=\left[x_{t},y_{t},z_{t},vx_{t},vy_{t},vz_{t}\right]$. To estimate this state, the particle filter uses a set of *N* particles that represents different possible states. In Algorithm 1 a schematic overview of the particle filter is given. First we need to initialize the particles. The position of the particles is spread over an area of 5 m × 5 m, which was the size of our test area, and the initial velocity and acceleration of each particle was set to zero. Next, we update the state of each particle with a movement model, which we will specify in Section 4.1.1. In the following step, we give each particle a weight based on how likely it is that this particle is at the true position. In Section 4.1.2, we will specify how we determined these weights and once we have these weights, the position can be estimated as the position of the particle with the highest weight or as the weighted average over all particles. In this paper we use the particle with the highest weight. As the last step in our particle filter, we resample our particles. This means that we will choose a new particle set based on the old set, where a particle with a large weight has a higher chance of appearing more than once in this new set. Without resampling, more and more samples would be far from the true location, which would deteriorate the performance of the particle filter. Resampling tries to limit this decrease in performance by focusing more on particles with a large weight, and hence keeping most particles close to the true position. In the next sections, we will give some more details about the different steps of the particle filter algorithm and how they are implemented.

**Algorithm 1** Particle filter1:Initialize particles2:**while** true **do**3:    Update particles with the movement model (Section 4.1.1)4:    Determine weight for every particle based on the UWB measurement (Section 4.1.2)5:    Return the particle with highest weight6:    Resample (Section 4.1.3)7:**end while**


#### 4.1.1. Movement Model

To model the movement of our particles, we will use a discrete time white noise acceleration model for $x_{t}$:

(1) $$x_{t+1}=Fx_{t}+{Gn}_{a}$$

(2) $$F=\left[\begin{array}{cccccc} 1 & 0 & 0 & \Delta T & 0 & 0\\ 0 & 1 & 0 & 0 & \Delta T & 0\\ 0 & 0 & 1 & 0 & 0 & \Delta T\\ 0 & 0 & 0 & 1 & 0 & 0\\ 0 & 0 & 0 & 0 & 1 & 0\\ 0 & 0 & 0 & 0 & 0 & 1 \end{array}\right]$$

(3) $$G=\left[\begin{array}{ccc} \frac{\Delta T^{2}}{2} & 0 & 0\\ 0 & \frac{\Delta T^{2}}{2} & 0\\ 0 & 0 & \frac{\Delta T^{2}}{2}\\ \Delta T & 0 & 0\\ 0 & \Delta T & 0\\ 0 & 0 & \Delta T \end{array}\right]$$

where $x_{t+1}$ is the new state at timestamp t+1, $x_{t}$ is the old state at timestamp *t*, and ΔT is equal to the time difference between timestamps *t* and t+1. In this model, acceleration $n_{a}$ is modelled as Gaussian noise with standard deviation $\sigma_{a}$, which can be used to limit how much the acceleration can change between two timestamps.

#### 4.1.2. Determine Weight

Once we have updated all particles according to our movement model, we will give each particle a weight based on the UWB measurements at that timestamp. We will assume that the ranging errors have a Gaussian distribution (4), where μ is the measured range, σ is the standard deviation of the ranging error, and *x* is the range between the particle and the anchor. From our measurements we obtained a value for σ of approximately 250 mm. The total weight of each particle can then be calculated with Equation (5), where n∈[1,N], N is the number of particles, M is the number of available measurements, $x_{n,i}$ is the distance between the *n*th particle and the anchor corresponding with the *i*th measurement, and $\mu_{i}$ and $\sigma_{i}$ are respectively the measured range for the *i*th measurement, and the standard deviation for the *i*th measurement.

(4) $$f\left(x|\mu ,\sigma \right)=\frac{1}{\sigma \sqrt{2\pi }}\exp\frac{-{\left(x-\mu \right)}^{2}}{2\sigma^{2}}$$

(5) $$w_{n}=\prod_{i=1}^{M}f(x_{n,i}|\mu_{i},\sigma_{i})$$

#### 4.1.3. Resampling

In a particle filter without resampling, particles tend to diverge from the real position of the user, which eventually results in many particles with very low weight. Hence, it is useful to put more particles close to the real position. From the old particle set, the resampler will pick the same number of particles. Some particles might be chosen more than once, as particles with a high weight have a higher chance of being picked more than once. Multiple resampling algorithms exist, e.g., multinomial, stratified, and systematic resampling. In this work we chose the multinomial resampler. A disadvantage of resampling is that if the UWB range has a large error due to the absence of LoS, particles far from the real position may obtain a high weight so that most particles after resampling will also be far from the real position. To solve this problem, we can use a random position for a fixed percentage of the particles.

### 4.2. Kalman Filter

Besides particle filters, the use of Kalman filters is often proposed. As with the particle filter, we will model our system with the same state $x_{t}=\left[x_{t},y_{t},z_{t},vx_{t},vy_{t},vz_{t}\right]$. The Kalman filter consists of two phases, i.e., the prediction phase and the measurement phase. In the prediction phase, the filter estimates the new state based on the old state and a movement model. We will use the same movement model (Equations (1)–(3)) that we used for the particle filter so that we can compare both filters. In the measurement phase, UWB ranges for different anchors are used to update the estimate of the state $x_{t}$. These measurements have the following form:

(6) $$d_{i}=\sqrt{{\left(x_{t}-a_{x,i}\right)}^{2}+{\left(y_{t}-a_{y,i}\right)}^{2}+{\left(z_{t}-a_{z,i}\right)}^{2}}+n_{d},$$

where $d_{i}$ is the measured range between the *i*th anchor and the tag, $n_{d}$ is the noise on the range measurement (which we assume as Gaussian with a standard deviation σ of 250 mm as in Section 4.1.2), $x_{t}$, $y_{t}$ and $z_{t}$ are the current estimates of the position in our state, and $a_{x,i}$, $a_{x,i}$ and $a_{x,i}$ are respectively the *x*, *y*, and *z* component of the $i^{th}$ anchor. Note however, that Equation (6) is not linear, which implies that an EKF or UKF must be used. In this work, we will consider only an EKF implementation.

Compared to state-of-the-art Kalman filters, the use of particle filters in the context of sport activities has several advantages: (1) The particle filter can include non-Gaussian noise models, which are more realistic in challenging sport conditions; and (2) particle filters can include sport-specific domain knowledge about likely paths (e.g., runner tracks or bicycle lanes) or likely player areas (e.g., handball regions, …). Such constraints can easily be added to particle filters while this would be more difficult for a Kalman filter. Nevertheless, we include the Kalman filter also to allow fair comparisons.

### 4.3. Results

**Figure 9:**
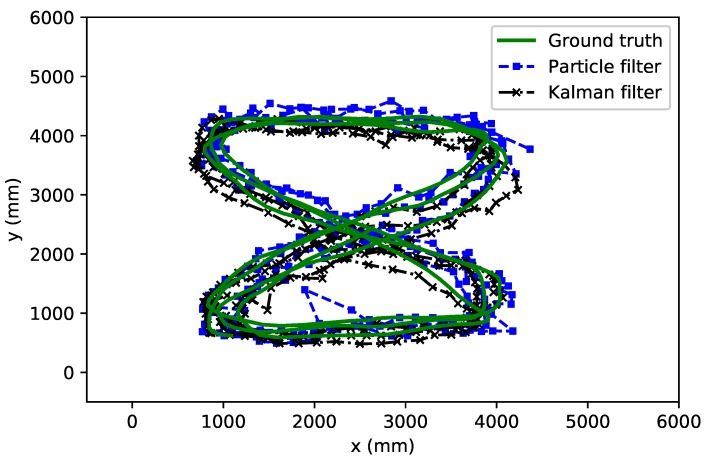
Position computed with particle filter (dashed line with square marker), with the Kalman filter (dashed line with cross marker), and with ground truth (solid line) for an eight-shaped path.

**Figure 10:**
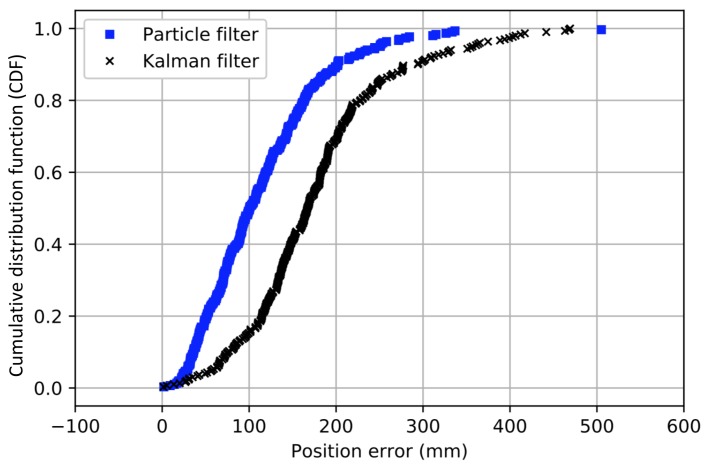
Cumulative distribution of the UWB positioning error for an eight-shaped path using the particle filter (squares), and the Kalman filter (crosses).

To evaluate the performance of our particle and Kalman filter we traversed an eight-shaped path while collecting UWB measurements with the tag, and simultaneously tracking the ground truth with the camera system. In our tests we set the number of particles *N* equal to 1000. In Figure 9, we show the traversed path as estimated with the particle filter as well as with the Kalman filter, together with the ground truth obtained from the MOCAP system. From this figure, we can clearly see that both the particle and Kalman filter are able to follow the correct path. In Figure 10, we show the cumulative distribution of the position error reported by both filters. We can see that for the particle filter, most errors are well below 30 cm and that 50% of the time the error is smaller than 10 cm. The Kalman filter on the other hand seems to have a lower accuracy as 50% of the time the error is lower than 17 cm. In order to try to improve our filters, we will consider different possible optimizations in the following sections.

## 5. Localization Results in Large-Scale Scenarios

**Figure 11:**
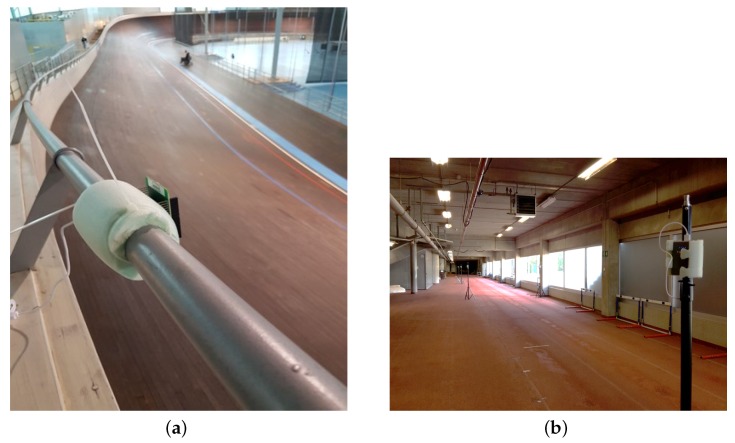
(**a**) Vlaams Wielercentrum Eddy Merckx; and (**b**) the Sports Arena Gent VZW.

Although the used MOCAP system can not be used in large areas, we visually evaluate the system also in two large-scale indoor sport environments, namely for cycling and running. The experiments took place in two facilities, i.e., Vlaams Wielercentrum Eddy Merckx (Figure 11a) and the Sports Arena Gent VZW (Figure 11b), both located in Gent, Belgium.

The UWB settings are the same as those listed in Table 1. For each of the sports, we explain how the athletes were tracked and afterwards we visualize preliminary localization results, which shows that even in these large-scale conditions UWB can be successfully used to track athletes.

### 5.1. Indoor Cycling

**Figure 12:**
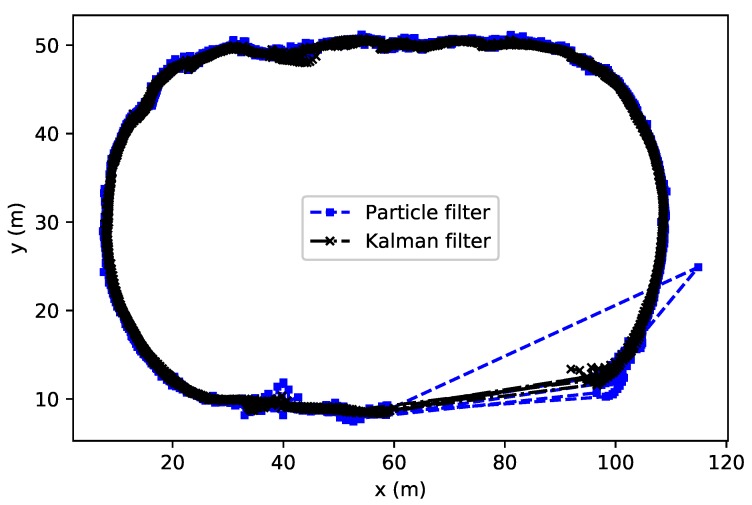
UWB tracking for indoor cycling with particle and Kalman filters.

The Vlaams Wielercentrum Eddy Merckx is an arena for track cycling. The track follows Olympic and World Championship rules, measuring 250 m. The goal is to track the cyclists going around the track during their training and competitions. To provide coverage for the entire track, 28 anchor nodes were needed. The cyclist wore the mobile tag on the helmet since this was an easy deployment location with little to no impact on the cyclist performance. The reference lap time was 27 s, resulting in an approximate speed of 9 m/s. We applied the same two algorithms presented in Section 4. In Figure 12, the cyclist’s path is clearly shown. Particle and Kalman filters are comparable with the only exception that in the bottom-right part of the graph, Kalman is more precise. It is worth mentioning that in this small sector we had to deal with a limitation due to software design of the UWB boards we used. In fact, a maximum of 20 anchors could be used at the time and therefore, some positioning data is missing. However, the Kalman filter seems to recover faster than the particle filter, which also shows some outliers.

### 5.2. Indoor Running

Secondly, infrastructure was deployed to track indoor runners on the training track of the Sports Arena Gent VZW. More precisely, a 60-m, 3-lane straight track normally used to train for specific acivities such as sprints and steeplechase. The number of deployed anchors was decreased to 10 and the main difference with the previous case was that the tag was worn by the runner on his side arm, which was the third best option in terms of packet loss and accuracy according to the evaluation in Figure 4 and Figure 7. Although the arms move more significantly during the run with respect to the center of mass of the body, this location ensures the best comfort for the runner.

**Figure 13:**
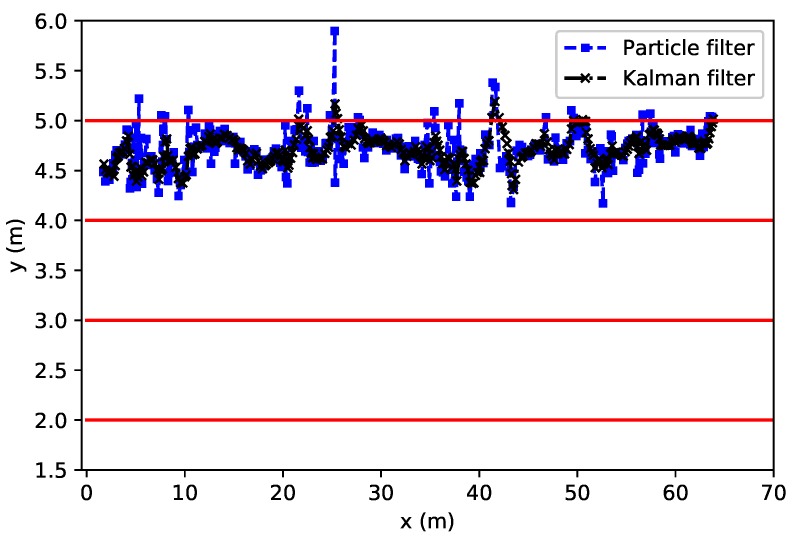
UWB tracking for indoor running with particle and Kalman filters in a 3-lane track.

Figure 13 demonstrates that the runner is clearly positioned within the first lane (4 m < y < 5 m). The estimates are slightly biased towards the upper line since the tag is located on the arm. However, even in this case, the algorithms allow for precise tracking of the runner. Nevertheless, the particle filter seems less precise and estimates are sometimes more spread out than with Kalman. Future work will compare these results with a gold standard system to evaluate the positioning error in the same way we did for the lab measures in dynamic conditions, for which we will introduce several algorithmic optimizations to improve the accuracy of the system.

## 6. Advanced Accuracy Improving Optimizations

The algorithm designed in Section 4 computes the position of the mobile tag by applying a particle or Kalman filter algorithm, resulting in already very accurate results. To improve the accuracy even further, we propose and analyze three different optimizations: bias removal, NLoS detection, and path determination. We use three different movements to test our optimizations, i.e., running at a constant velocity, walking at a constant velocity, and accelerating back and forth on a straight line. The mean absolute error (averaged over multiple tests for each movement) for each optimization is given in Table 4 for the particle and Kalman filters, respectively.

### 6.1. Removing Bias

**Figure 14:**
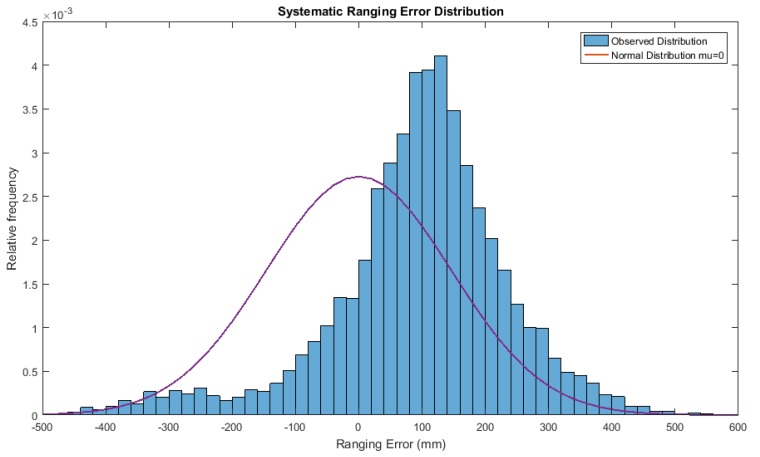
Systematic ranging error distribution.

During our measurements, we noticed that even in the best scenario, i.e., static and LOS conditions such those in the first experiment of Section 3, the estimated distances to the fixed anchor were somehow systematically shifted from the ideal scenario, which was probably caused by poor antenna calibration procedure [19]. From Figure 14, wherein the distribution of ranging errors for different distances is shown, it can be seen that this shift equals approximately 96 mm.

As this bias remains constant over all the measurements and through all the experiments regardless of the real distance, the first optimization simply consists in bias removal, which in this case is a simple subtraction of the computed bias.

The impact of this optimization is shown in Table 4, which considers three different movements patterns. The bias removal significantly improves the accuracy, decreasing the position estimate error with the particle filter in every scenario by at least 27.5 mm with a maximum improvement of 31% with respect to the case without optimizations. For the Kalman filter we see similar results, but in general the bias compensation has more of an effect for the particle filter.

### 6.2. NLoS Detection

**Figure 15:**
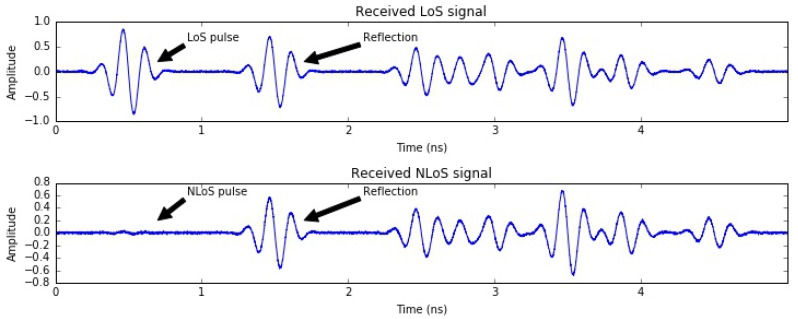
Received UWB pulses in LoS and NLoS conditions.

As we discussed in Section 3, the placement of the tag impacts the accuracy and availability of the anchors as the human body obstructs the UWB signal. Therefore, a deeper investigation of what this implies is needed. Having the tag on the back most likely creates an obstruction of the anchor placed just in front of the body. We showed that in this specific case the anchor is not in range at all, resulting in high packet loss, i.e., low anchor availability. On the other hand, some anchors will operate in NLoS conditions, wherein the tag can not see the anchor directly, but they can still successfully exchange packets, for example from signals reflected by walls or obstacles. Ideally, these NLoS measurements should be excluded since the traversed distance will be larger than the actual distance between tag and anchor. To overcome this problem, we use a simple mechanism to detect NLoS measurements exploiting the nature of UWB signals. High timing resolution is probably the most relevant characteristic of UWB and an example of received pulses is shown in Figure 15.

Consequently, it is possible to estimate both the receive power level ($RX_{power}$) and the power for the first path signal ($FP_{power}$). These power levels can be computed respectively as follows [11]:

(7) $$RX_{power}=10\times log_{10}\left(\frac{C\times 2^{17}}{N^{2}}\right)-A\left[dBm\right]$$

(8) $$FP_{power}=10\times log_{10}\left(\frac{{F_{1}}^{2}+{F_{2}}^{2}+{F_{3}}^{2}}{N^{2}}\right)-A\left[dBm\right]$$

where *C* is the channel impulse response, *A* is the pulse repetition frequency (PRF)-dependent constant, *N* is the preamble accumulation count value, and $F_{i}$ is the *i*th path amplitude. These parameters are provided by the Decawave chip and we used them to compare the two power levels (Equations (7) and (8)). We decide that LoS is absent if $RX_{power}-FP_{power}>10$ dB. When the difference is <6 dB, we can positively consider LoS conditions, while when 6 dB $<RX_{power}-FP_{power}<$ 10 dB, it is not possible to classify the communication with high certainty and LoS conditions are assumed although that might not be the case. Based on the difference between the first path and received power level we will use a different value σ in Equation (4) to determine the weights for each particle for the particle filter or use a different value σ in Equation (6) for the Kalman filter. For NLoS measurements, this value is larger than for the LoS measurements, indicating that these measurements are less reliable and hence should have less of an influence on estimating the position. Dedicated research has been performed on NLoS detection, e.g., applying machine learning and training the system [20]. However, as the NLoS recognition is not the focus of this paper, we refer to the aforementioned work for more advanced LoS classifiers. When applied to our localization algorithms, determining whether the communication happened in LoS or NLoS conditions only has a small influence on the accuracy of estimated position, as shown in Table 4, mainly improving the accuracy of the running case with constant movement. The limited accuracy gains are most likely due to the specific setup of the experiments in the middle of a large room with very few obstacles. We expect the impact of LoS detection to increase when applied to sporting scenarios in smaller areas with more obstacles and reflections.

### 6.3. Path Determination

In Section 3, we showed that some human body positions have better performance in terms of packet loss than others. However, even if the packet loss is minimized, there will still be packets that are corrupted. Packet loss is directly related to the last improvement we present in our paper: the path determination. When the tag is moving, it is a good practice to spread the particles according to the speed and direction of the tag itself. The same is true for the Kalman filter, i.e., a model that tries to predict the movement more precisely than just a random movement (Equations (1)–(3)) should give a higher accuracy. The distance shift is computed using the last estimated velocity and the current time difference (Equations (8) and (9)).

(9) $$\Delta \;x_{n}=\Delta t_{n}\frac{\Delta x_{n-1}}{\Delta t_{n-1}}=\Delta t_{n}\frac{\sqrt{{\left(x_{n}-x_{n-1}\right)}^{2}+{\left(y_{n}-y_{n-1}\right)}^{2}}}{\Delta t_{n-1}}$$

(10) $$\alpha_{n}=\arctan\frac{y_{n}-y_{n-1}}{x_{n}-x_{n-1}}$$

where Δ*t*${}_{n}$ is the time difference between timestamps n+1 and *n* in seconds, Δ*t*${}_{n-1}$ is the time difference between timestamps *n* and n−1 in seconds, and x${}_{n}$, y${}_{n}$ are the last estimation of the coordinates (m). In Table 4, we can see that the path determination did not have a large impact on the mean absolute error for either of the localization algorithms. One reason for the particle filter might be that the number of particles is large enough so that it is not necessary to move the particles in a certain direction and that a random spread of the particles leads to the same performance. Another reason for both filters might be that errors in the position derived from the UWB ranges result in an erroneous estimation of the current heading direction, which moves the estimated particles or position in the wrong direction. To predict the path more accurately, more than one location estimate in the past might useful.

## 7. Conclusions

Location-based information requires accurate position estimates in order to provide meaningful results. UWB can be considered as one of the most promising technologies for obtaining centimeter-level position estimates. In the last few years, a lot of effort has been dedicated to investigating the UWB standard. Recently, with the commercialization of affordable UWB chips, this technology has gained even more popularity. In our research we intended to explore a less studied aspect of UWB to allow the possibility for use in sporting purposes, e.g., tracking athletes’ speed. Initially, we studied and evaluated the ideal placement of the mobile tag to be tracked on the human body. We investigated both the impact of the posture and tag position on ranging errors. The resulting best body part for the positions tested, both in terms of packet loss and anchor visibility (due to body obstruction and reflection), was the head of the athlete. This was because it resulted in less interference between the body and the fixed nodes, although the side of the arm and neck positions were also shown to be good alternatives. The second phase of the investigation focused on the design of a localization algorithm using data we gathered from real-life measurements. Various activities were tested, e.g., running and walking at constant speed, accelerating, and jumping. The output of the particle and the Kalman filter algorithm showed encouraging results, with an average error of around 20 cm when the tag was placed on the head. Although both algorithms showed similar performance, an additional benefit of the particle filter compared to the Kalman filter was that non-Gaussian noise models and sport-specific path or area constraints were easily added.

Finally, several optimizations were proposed to further increase the accuracy by more than 30%. The outcomes also demonstrate that the choice of which optimization to include depends strongly on the type of localization algorithm that is used, as well as the type of activity that is tracked. This shows that there is room for even further improvement by designing accuracy enhancements for specific activities or sport types.

To summarize, we have shown that UWB is a good candidate for the sports analysis and tracking market. Simple but effective enhancements can help the system to overcome certain technology limits and improve overall performance.

## Figures and Tables

**Table 1:** Experimental overview. UWB radio settings, UWB tag positions on the human body, Postures and dynamic movements tested.

**UWB Radio Settings**	Channel 1
	6.81 Mbit/s bitrate
	1024 symbol preamble length
	64 MHz pulse repetition frequency (PRF)
**On-Body Tag Placement Positions**	Chest
	Stomach
	Hip
	Side (right) arm
	Front (right) arm
	Side (right) upper leg
	Front (right) upper leg
	Neck
	Head
**Postures and Dynamic Movements**	Flat: User lying on the floor in a prone position
	Bent over: User bent over his leg
	Full squat: User bent on his knees in a lower position so that he is almost sitting on his heels
	Up: User standing still
	Jumping: User jumping up and down on the same spot (0.5 jumps/s)
	Walking: User walking at a constant speed (1.7 m/s)
	Running: User running at a constant speed (3.0 m/s)
	Accelerating: User constantly accelerating

**Table 2:** Packet loss (%) per anchor depending on tag placement.

	Anchor 0	Anchor 1	Anchor 2	Anchor 3	Anchor 4	Anchor 5	Anchor 6	Anchor 7	Average Packet Loss
**Chest**	0.00	0.00	5.60	53.20	79.20	70.40	0.40	1.20	26.25
**Stomach**	0.00	0.80	10.00	17.20	29.60	18.00	0.40	0.80	9.60
**Hip**	4.40	9.20	92.00	4.40	2.40	8.00	27.20	1.60	18.65
**Side arm right**	0.80	4.40	38.00	2.40	9.20	1.60	0.80	1.20	7.30
**Front arm right**	0.80	0.40	10.40	12.80	56.40	3.60	0.40	2.00	10.85
**Side right upper leg**	0.40	5.20	9.20	1.20	0.80	4.80	1.20	0.40	2.90
**Front right upper leg**	0.80	6.00	2.80	7.60	16.40	10.00	0.80	0.40	5.60
**Back of the neck**	7.20	46.00	4.00	1.60	1.20	0.40	2.40	13.60	9.55
**Head**	0.80	0.80	1.20	2.40	4.80	0.00	0.40	1.20	1.45

**Table 3:** Impact of different postures and activities on packet loss while ranging with eight anchors.

Position	Posture/Activity	Ranging Failures
**Side Arm**	Flat	8%
	Bent Over	28%
	Full Squat	18%
	Up	34%
**Neck**	Flat	3%
	Bent Over	25%
	Full Squat	6%
	Up	32%
**Head**	Flat	11%
	Bent Over	19%
	Full Squat	19%
	Up	8%
	Acceleration	17%
	Jumping	14%

**Table 4:** Mean absolute error (MAE) when using algorithmic optimizations for the head position case, with the particle filter, and the Kalman filter. NLoS: non-line-of-sight.

Algorithm	Movement	Without Optimization	Bias Compensation	NLoS Detection	Path Determination
Particle filter	Running (v = const)	193.9 mm	132.2 mm	189.4 mm	187.6 mm
	Walking (v = const)	204.6 mm	169.0 mm	208.8 mm	198.4 mm
	Accelerating	151.0 mm	123.5 mm	152.5 mm	160.8 mm
Kalman filter	Running (v = const)	179.3 mm	140.5 mm	166.4 mm	215.7 mm
	Walking (v = const)	203.8 mm	170.3 mm	207.6 mm	213.4 mm
	Accelerating	166.7 mm	149.4 mm	164.3 mm	153.9 mm

## References

[1] Location-Based IoT and Geo Analytics Market Outlook and Forecasts 2017–2022. http://www.marketsandmarkets.com/Market-Reports/location-analytics-market-177193456.html.

[2] Van Haute T., De Poorter E., Lemic F., Handziski V., Wirström N., Voigt T., Wolisz A., Moerman I. (2015). Platform for benchmarking of RF-based indoor localization solutions. IEEE Commun. Mag..

[3] Floyd R.E. (2015). RFID in Animal-Tracking Applications. IEEE Potentials.

[4] Sport Science Laboratory—Jacques Rogge of Ghent University. https://www.ugent.be/ge/bsw/en/sportlab.

[5] Oqus Qualisys Camera System. http://www.qualisys.com/cameras/oqus/.

[6] Liu H., Darabi H., Banerjee P., Liu J. (2007). Survey of wireless indoor positioning techniques and systems. IEEE Trans. Syst. Man Cybern. Syst. Part C Appl. Rev..

[7] Koyuncu H., Yang S.H. A survey of indoor positioning and object locating systems. Proceedings of the International Conference on Innovations in Information Technology.

[8] Zhu J., Chen Z., Luo H., Li Z. RSSI Based Bluetooth Low Energy Indoor Positioning. Proceedings of the International Conference on Indoor Positioning and Indoor Navigation.

[9] Lee J.Y., Scholtz R.A. (2012). Ranging in a dense multipath environment using an UWB radio link. IEEE J. Sel. Areas Commun..

[10] Leser R., Baca A., Ogris G. (2011). Local Positioning Systems in (Game) Sports. Sensors.

[11] Decawave ScenSor DW1000 Chip. https://www.decawave.com/products/dw1000.

[12] Milici S., Esposito A., Staderini E.M. (2017). Evaluating Athletic Performances with a Real Time Location and Tracking System. Mater. Sci. Forum.

[13] Djaja-Josko V., Kolakowski J. UWB positioning system for elderly persons monitoring. Proceedings of the Telecommunications Forum Telfor (TELFOR).

[14] Maalek R., Sadeghpour F. (2016). Accuracy assessment of ultra-wide band technology in locating dynamic resources in indoor scenarios. Autom. Constr..

[15] Perrat B., Smith M.J., Mason B.S., Rhodes J.M., Goosey-Tolfrey V.L. (2015). Quality assessment of an UWB positioning system for indoor wheelchair court sports. SAGE J..

[16] Pozyx Labs. https://www.pozyx.io/.

[17] Kumpuniemi T., Hamalainen M., Yazdandoost K.Y., Iinatti J. (2017). Human Body Shadowing Effect on Dynamic UWB On-Body Radio Channels. IEEE Antennas Wirel. Propag. Lett..

[18] Ledergerber A., D’Andrea R. Ultra-wideband range measurement model with Gaussian processes. Proceedings of the IEEE Conference on Control Technology and Applications (CCTA).

[19] APS011 Application Note Sources of Error in DW1000 Based Two-Way Ranging (TWR) Schemes. http://www.decawave.com/sites/default/files/resources/aps011_sources.of_error_in_twr.pdf.

[20] Savic V., Larsson E.G., Ferrer-Coll J., Stenumgaard P. (2016). Kernel methods for accurate UWB-based ranging with reduced complexity. IEEE Trans. Wirel. Commun..

